# Targeting the mitochondrial apoptotic pathway: a preferred approach in hematologic malignancies?

**DOI:** 10.1038/cddis.2014.61

**Published:** 2014-03-06

**Authors:** K Brinkmann, H Kashkar

**Affiliations:** 1 Centre for Molecular Medicine Cologne (CMMC), Cologne Excellence Cluster on Cellular Stress Responses in Aging-Associated Diseases (CECAD), Köln, Germany; 2 Institute for Medical Microbiology, Immunology and Hygiene (IMMIH), University of Cologne, Köln, Germany

**Keywords:** apoptosis, BH3 mimetics, Bcl-2 targeting

## Abstract

Acquired resistance toward apoptosis represents one of the hallmarks of human cancer and a major cause of the inefficacy of most anticancer treatment regimens. Based on its ability to inhibit apoptosis, the B-cell lymphoma/leukemia 2 (Bcl-2) protein family has garnered the most attention as a promising therapeutic target in cancer. Accordingly, efforts have lately been focused on the development of drugs targeting Bcl-2 proteins with considerable therapeutic success, particularly in hematologic malignancies. Here, we review the previous studies and highlight the pivotal role of the Bcl-2 protein family in the homeostasis of hematologic tissue compartment. This knowledge provides more insight into why some cancers are more sensitive to Bcl-2 targeting than others and will foster the clinical evaluation of Bcl-2-targeting strategies in cancer by avoiding severe on-target side effects in the development of healthy tissues.

## Facts

- Inefficient mitochondrial apoptosis is a key determinant in therapeutic success of a number of anticancer regimens.
- Mitochondrial apoptosis is tightly controlled by the Bcl-2 protein family.
- Bcl-2 proteins have a crucial role in the development and the homeostasis of cells of hematopoietic origin.
- Imbalanced expression of Bcl-2-family members has been readily associated with the development of hematologic malignancies such as lymphoma, leukemia or myeloma.
- Several small-molecule inhibitors of Bcl-2 proteins have been developed and are currently under clinical evaluation with a marked susceptibility to hematologic malignancies.

## Open Questions

- What is the impact of Bcl-2-targeting anticancer therapy in solid tumors?
- How can hematologic side effects be avoided upon Bcl-2-targeting anticancer therapy?
- How and in combination with which additional chemotherapeutics provoke Bcl-2-antagonizing protocols for a potent anticancer effect?

Compelling studies conducted over the last 20 years established the concept that apoptosis serves as a natural barrier to cancer development, which is triggered autonomously during the process of malignant transformation or as a result of anticancer treatment.^1^ Accordingly, acquired resistance toward apoptosis is a hallmark of most types of human cancer and a major cause of the inefficacy of most anticancer treatment regimens.^2^ Mitochondria represents a central regulatory node in the apoptotic machinery and the decisive event thereby is the process of mitochondrial outer membrane permeabilization (MOMP). Upon MOMP, multiple pro-apoptotic molecules are released from the mitochondrial intermembrane space to coordinate most of the hallmarks of apoptosis, like nuclear condensation and caspase activation. Inefficient MOMP has been considered to be one of the key determinants of therapeutic success of a number of anticancer regimens. Accordingly, reactivation of the mitochondrial apoptotic machinery by restoration of MOMP has been viewed as a promising strategy to combat human cancer.^1^

## Bcl-2 Protein Family, the Gatekeepers of Mitochondrial Outer Membrane

**Figure 1:**
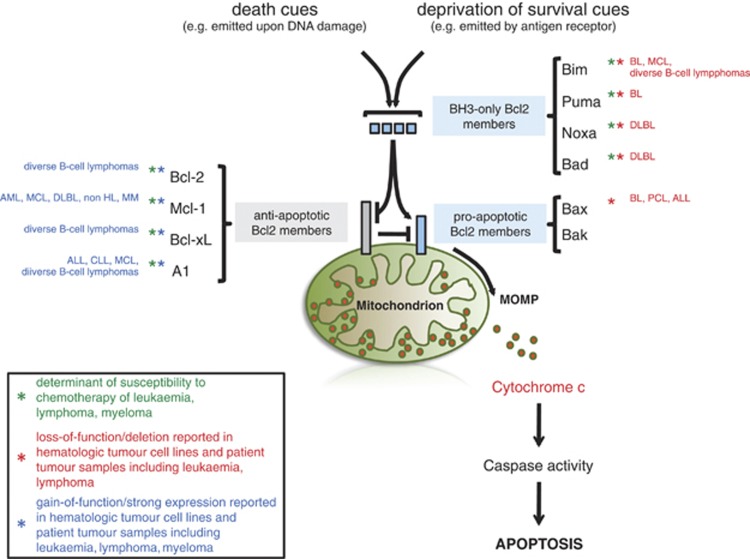
Bcl-2 protein family in apoptosis and hematologic malignancies. Mitochondria represent a cellular regulatory node in apoptosis induced by death cues (e.g. DNA damage) or by the deprivation of survival signals (e.g. emitted by antigen receptor). The pro-apoptotic activity of mitochondria involves the mitochondrial outer membrane permeabilization (MOMP), which is tightly regulated by anti- and pro-apoptotic Bcl-2 protein family members. This is in turn is controlled by BH3-only proteins that initiate MOMP by either direct binding to the pro-apoptotic Bcl-2 members or by antagonizing their anti-apoptotic counterparts. Upon MOMP cytochrome *c* is released from the mitochondrial intermembrane space and initiates proteolytic activation of caspases, culminating in apoptotic cell death. Imbalanced expression of Bcl-2-family members has been readily associated with the development of hematologic malignancies such as lymphoma, leukemia or myeloma as summarized. PCL, plasma cell leukemia. Astrisks indicate the association of the Bcl-2 protein family members (gain- or loss-of-function) in chemosusceptibility (green) and/or malignant transformation of lymphoid malignancies (red or blue)

MOMP is tightly controlled by the Bcl-2 (B-cell lymphoma/leukemia 2) protein family via protein–protein interaction (Figure 1). Bcl-2-family members have been grouped into three classes (Figure 1) The antiapoptotic Bcl-2-family members including Bcl-2, Bcl-xl and Mcl-1 inhibit apoptosis, whereas a second class including Bax and Bak promotes apoptosis. A third divergent class of BH3-only proteins including Bad, Bik, Bid, Bim, Bmf, Noxa and Puma have a conserved BH3 domain that can bind and regulate the activity of Bcl-2 proteins. Recent evidence suggests that BH3-only proteins derepress and liberate Bax and Bak by direct binding and inhibition of anti-apoptotic family members including Bcl-2. By contrast, an opposing model postulates direct activation of Bax and Bak by BH3-only proteins including Bim, tBid and Puma (Figure 1).^1^

## Apoptosis Represents a Fundamental Regulatory System During Hematopoiesis

Hematopoiesis gives rise to blood cells of different lineages throughout normal life. Abnormalities in this developmental program lead to blood cell diseases including leukemia and lymphoma.^3^ During hematopoiesis, a complex interacting network of cytokines and adhesion molecules tightly regulates the survival of progenitor cells, both positively and negatively. Following deprivation of these survival cues apoptotic death of progenitor cells actively safeguards hematologic homeostasis and prevents malignant transformation.^4^ Accordingly, almost 90% of pre-T- and B-cells undergo apoptosis during maturation in the thymus or bone marrow, respectively. Furthermore, after antigen exposure T- and B-cells undergo clonal expansion, giving rise to the generation of a large number of active effector lymphocytes. Apoptosis triggers the shutdown of the immune response when an infection has been overcome.^5^ Importantly, key elements of the basic apoptotic signaling machinery have been first discovered in the hematopoietic system associated with diseases when aberrantly expressed (Bcl-2 and lymphoma) or mutated (CD95 and ALPS),^6^ underscoring the intimate association of the apoptotic machinery, in particular, Bcl-2 proteins with the homeostasis of the hematopoietic system (Figure 1).

## Bcl-2 Proteins – Their Physiologic Role in Cells of Hematopoietic System and Hematologic Cancer

**Figure 2:**
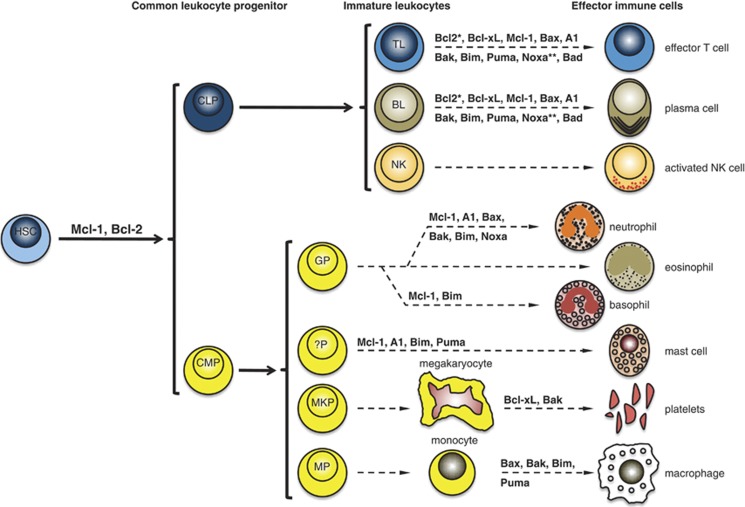
The Bcl-2 protein family in the development and homeostasis of the hematologic system. A summary of the current knowledge about the physiological role of Bcl-2 protein family in hematopoiesis based on the results obtained in mice. common lymphoid progenitor (CLP), common myeloid progenitor (CMP), T lymphocyte (TL), BL (B lymphocyte), NK (natural killer cells), GP (granulocyte progenitor), ?P (unknown progenitor), MKP (megakaryocyte progenitor), MP (monocyte progenitor). *Bcl-2 ablation reduces the number and the lifespan of leukocytes but presumably does not impact on lymphoid development. **Noxa impacts on the lymphocyte function upon infection but is not involved in lymphoid development

**Figure 3:**
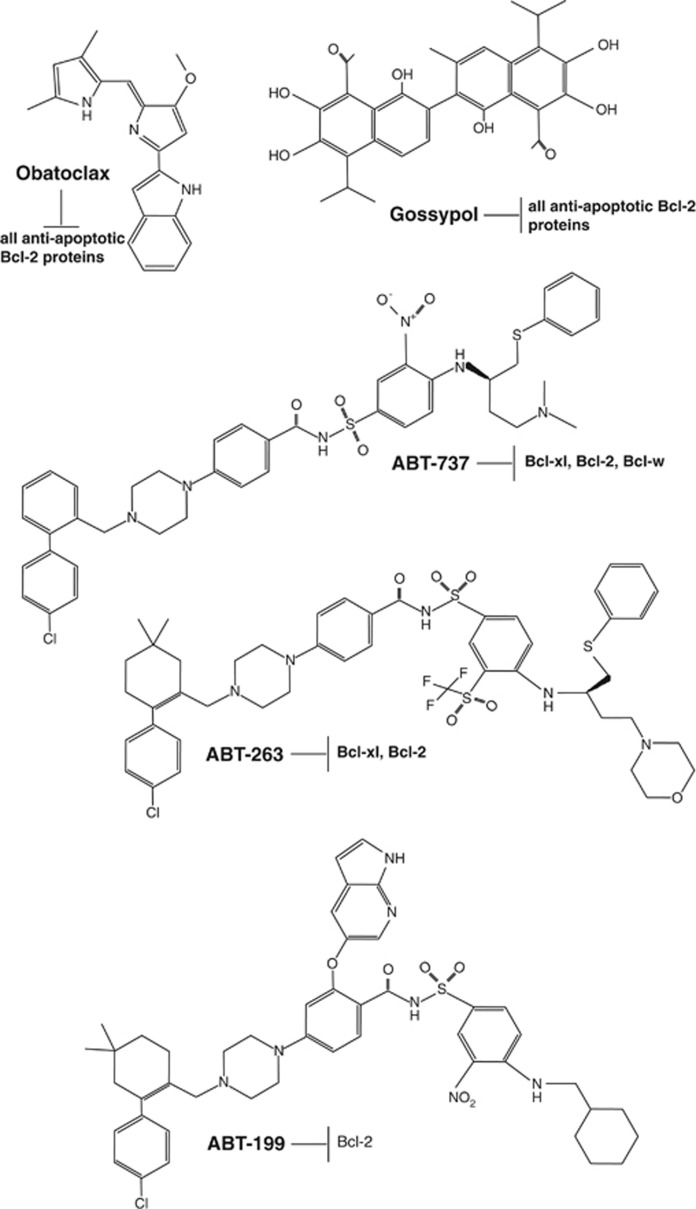
Structural view of BH3 mimetics. *Obatoclax* (GX15-070) is a Bcl-2 homology domain-3 (BH3) mimetic. It occupies a hydrophobic cleft within the BH3-binding groove of Bcl-2, antagonizing Bcl-2 and thus inducing apoptosis. Gossypol is a natural phenol derived from the cotton plant (genus: *Gossypium*). The phenolic aldehyde permeates cells and acts as an inhibitor for several dehydrogenase enzymes and in particular in its (*R)-*configuration (known as AT-101) it acts as a pan-Bcl-2-family inhibitor, capable to bind and inhibit most antiapoptotic Bcl-2-family members. Abt-737 is a small-molecule BH3 mimetic developed by Abbott that binds to the hydrophobic BH3-binding groove of antiapoptotic Bcl-2-family members. ABT-737 binds with high affinity (Ki ≤1  nM) to Bcl-xl, Bcl-2 and Bcl-w, but not to the less-homologous proteins Bcl-b, Mcl-1 and A1. Abt-263 (Navitoclax, Abbott Laboratories) is structurally related to Abt-737. It represents an orallybioavailable small-molecule Bad-like BH3 mimetic which efficiently antagonizes antiapoptotic Bcl-2-family members (Ki's of <1 nM for Bcl-2, Bcl-xl and Bcl-w). Abt-199, generated by Abbott is a high-affinity Bcl-2-selective small-molecule BH3 mimetic. It is not interacting with Bcl-xl and thus not interfering with platelet homeostasis

Imbalanced expression of Bcl-2-family members has been readily associated with the development of hematologic malignancies such as human lymphoma, leukemia or myeloma. Besides the extensive biochemical characterization, gene-targeting experiments in mice repeatedly showed that Bcl-2 proteins are essential for the development and homeostasis of the hematopoietic system. In the following we will summarize the data obtained in the previous years demonstrating the pivotal role of Bcl-2 proteins in hematologic compartment homeostasis (Figure 2), which may account for the observed association of hematologic malignancies with imbalanced Bcl-2 expression (Figure 1) and the marked susceptibility of hematologic malignancies toward Bcl-2-targeting strategies (Figure 3 and Table 1).

## Antiapoptotic Bcl-2 Proteins

The discovery of Bcl-2 family of proteins is intimately linked to many B-cell malignancies.

The *bcl-2* gene was initially discovered at the t(14;18) chromosome translocation breakpoint in B-cell follicular lymphomas, where its transcription becomes excessively driven by the immunoglobulin heavy chain gene promoter and enhancer on chromosome 14.^7^ In line with the data obtained in human tumor samples, mice lacking *bcl-2* have severe defects in the development of lymphoid progenitor cells from hematopoietic stem cells (HSC) and display reduced lifespan of lymphoid and myeloid cells.^8, 9, 10^ Conversely, early studies reported that Bcl-2 overexpression enhanced the survival of T-^11^ and B-cells.^12^ More strikingly, ectopic expression of Bcl-2 was capable of rescuing lymphopoiesis in SCID mice.^13^ The oncogenic potential of Bcl-2 was explored by showing that its overexpression facilitates the *c-myc*-driven proliferation of B-cell precursors and tumorigenesis.^14^

Myeloid cell leukemia sequence 1 (Mcl-1) was identified as an immediate-early gene induced by TPA-mediated differentiation of a human myeloid leukemia cell line (ML-1).^15^ *mcl-1* is one of the most highly amplified genes in a variety of human cancers. Specifically, elevated Mcl-1 was shown in acute myeloid leukaemia (AML),^16^ mantle cell lymphoma (MCL),^17^ diffuse large B-cell lymphoma (DLBL),^18^ non-Hodgkin's lymphoma^19^ and multiple myeloma (MM).^20^ In line with these observations, removal of Mcl-1 caused cell death of transformed AML and rescued AML-afflicted mice from disease development.^21^ Mcl-1 is unique among the antiapoptotic Bcl-2 members in being essential for early embryonic development. Deletion of *mcl-1* results in lethality at embryonic day 3.5,^22^ whereas tissue-specific ablation of *mcl-1* in mice demonstrated that Mcl-1 is essential for the survival and the development of B- and T-lymphocytes,^23^ germinal center formation and B-cell memory,^24^ plasma cells,^25^ neutrophils,^26^ basophil and mast cells,^27^ and has an obligate role for the survival of HSCs.^28^ Remarkably, inducible Cre-mediated deletion of even a single Mcl-1 allele substantially impaired the growth of *c-myc*-driven mouse lymphomas.^29^ In line with these observations, overexpression of Mcl-1 results in the development of lymphomas^30^ identifying Mcl-1 as a critical regulator of hematopoiesis and hematologic malignancies.

Initial studies using low stringency hybridization assays in chicken lymphoid cells identified *bcl-x*, a *bcl-2*-related gene that can function as a Bcl-2-independent regulator of apoptosis. Alternative splicing results in two distinct *bcl-x* mRNAs. The protein product of the larger mRNA, Bcl-xl, was similar in size and predicted structure to Bcl-2.^31^ Similar to Bcl-2 and Mcl-1, elevated Bcl-xl expression has been frequently observed in hematologic malignancies and is implicated to have a role in disease progression.^32^ *bcl-x*^*−/−*^ mice died at embryonic day 13 and displayed massive cell death of immature hematopoietic cells and thus severe defects in the development of the hematopoietic system,^33^ underlining the essential role of Bcl-x for the survival and development of lymphoid cells. In line with these observations, an independent approach showed that genetic ablation or pharmacological inactivation of Bcl-xl reduces platelet half-life and causes thrombocytopenia in mice.^34^ The central role of Bcl-xl in malignant transformation of hematopoietic cells was further strengthened with the fact that transgenic mice overexpressing Bcl-xl developed lymphomas.^35^

In contrast to Bcl-2 and Mcl-1 the described roles for A1 are more restricted. A1 is a hematopoietic tissue-specific gene that is expressed in several hematopoietic cell lineages, including T-helper lymphocytes, macrophages and neutrophils.^36^ High expression of *a1* mRNA was reported in acute lymphoblastic leukemia (ALL) and chronic lymphocytic leukemia (CLL), MCL and multiple types of DLBL,^37, 38, 39^ especially the OxPhos subgroup of DLBL.^40^ Mouse *a1* mRNA is induced during myeloid differentiation,^36^ mast cell activation upon an allergic reaction,^41^ lymphocyte development^42^ and lymphocyte and macrophage activation,^36^ emphasizing the importance of A1 in the hematopoietic system. Genetic deletions of *a1* in mice are challenging owing to the existence of multiple genetic copies of the *a1* gene in mice with a highly cell type-specific expression pattern. However, mice lacking only one *a1* gene, *a1-a*, show hematologic defects including reduced number of neutrophils^43^ and mast cells owing to enhanced apoptosis.^41^ A1 knockdown mice harboring a tg-driven short-hairpin RNA (shRNA) targeting *A1-a*, *A1-b* and *A1-d* mRNA did not show a severe phenotype. However, gene silencing appeared to be highly restricted and a significant knockdown of A1 abundance could only be achieved in thymocytes, while it was not efficient in mature lymphocytes.^44^ Remarkably, a tg shA1 mouse expressing a micro-RNA30-based precursor harboring a shRNA able to efficiently target all *A1* isoforms revealed multiple roles of A1 in lymphocyte development. Specifically, knockdown of *A1* impaired early stages of T-cell differentiation, B-cell homeostasis and sensitized transitional as well as follicular B-cells to apoptosis induced by ligation of the B-cell receptor. As a consequence, B-cell proliferation in response to mitogens was severely impaired, whereas T-cell survival was not affected. Moreover, granulocytes showed increased spontaneous death in culture or failed to accumulate in significant numbers *in vivo*.^45^ Enforced expression of A1-a in T-cells was found to accelerate T-cell survival in *a1-a tg* mice.^46^ Overexpression of A1 in B- and T-cell lineages in *Eμ-a1-a tg* mice extended the lifespan of thymocytes and early B-cells.^47^

## Pro-apoptotic Bcl-2 Proteins

Bax was initially identified as a Bcl-2-interacting factor capable to antagonize the pro-survival activity of Bcl-2.^48^ Based on a sequence homology, Bak (Bcl-2-homologous antagonist/killer) was identified and characterized as another pro-apoptotic member of the Bcl-2 protein family.^49, 50^ The mitochondrial apoptotic pathway converges on these two pro-apoptotic Bcl-2 proteins either of which is sufficient to drive MOMP in the majority of cells. Bax and Bak are highly redundant and this redundancy has been expected to limit their roles as tumor suppressors.^51^ However, different human malignancies including hematologic tumors exhibit mutated *bax*^52^ or *bak*^53^ and reduced expression levels of these pro-apoptotic Bcl-2-family members have been associated with a great number of hematologic malignancies including CLL, follicular lymphoma, MCL and marginal zone B-cell lymphoma.^54^ In line with these observations, genomic ablation of *bax* impaired *myc*-driven apoptosis and circumvents the selection of *p53* mutations during *myc*-mediated lymphomagenesis.^55^ Despite having an outwardly normal phenotype, *bax*^*−/−*^ or *bak*^*−/−*^ single knockout mice display a mild hematologic phenotype with only a mild lymphoid hyperplasia, probably owing to the functional redundancy of the two proteins.^56, 57^ Double-knockout *bak*^*−/−*^*bax*^*−/−*^ animals display an increase (three- to 10-fold) in both myeloid and lymphoid cells compared with either single *bak*^*−/−*^ *or bax*^*−/−*^ knockouts or wild-type mice. Progressive accumulation of mature B- and T-cells led to massive enlargement of the spleen and lymph nodes and infiltration of parenchymal organs.^57^ Furthermore, *bak*^*−/−*^*bax*^*−/−*^ mice display severe defects in the T-cell development and homeostasis after infection.^58^ Independently, inactivation of *bak* restored thrombocytopenia caused by *bcl-xl* deletion, suggesting a crucial role of Bak in platelet lifespan.^34^

## BH3-only Proteins

An expression screen for proteins that bind to Bcl-2 yielded a small novel protein, denoted as Bim, whose only similarity to any known protein was the short (9-amino-acid) BH3 motif shared by most Bcl-2 homologs.^59^ Sequence analysis of murine cDNAs revealed the presence of three major isoforms (BimEL-196aa, BimL-140aa and BimS-110aa), produced by alternative splicing.^59^ Independently, the same gene was discovered in an ovarian cDNA library, using Mcl-1 as bait, that they initially termed Bod (Bcl-2-related ovarian death agonist). Bim has a critical role during hematopoiesis (Figure 1) and the *in vivo* function of this protein has received the most attention among the pro-apoptotic Bcl-2 members. Blocking Bim expression by gene deletion or epigenetic silencing has a central role in the pathogenesis or the response to anticancer therapeutics in a number of human hematologic malignancies including Burkitt's lymphoma (BL), MCL and various B-cell non-Hodgkin's lymphomas.^60, 61, 62^ Consistent with these observations, gene-targeting experiments of the *bim* locus in mice demonstrated a crucial role of Bim in the homeostasis of most immune cells. In particular, *bim*^*−/−*^ mice have abnormal high numbers of T- and B-cells, macrophages and granulocytes owing to improved apoptosis resistance to several stimuli, including cytokine deprivation, abnormal calcium flux or irradiation.^63, 64^ They also show defects in the immune response shutdown as indicated by increased numbers of antibody-secreting plasma cells and the extended survival of activated cytotoxic T-cells after infection.^65, 66^ Furthermore, Bim seems to be essential for the apoptosis of autoreactive B- and T-cells (negative selection) resulting in systemic lupus erythematosus-like autoimmune disease in *bim*^*−/−*^ animals.^67, 68^ Moreover, loss of Bim accelerates lymphomagenesis in *Eμ-myc* transgenic mice.^69^

Puma (p53 upregulated modulator of apoptosis) is one of the most potent killers among the BH3-only proteins, which was initially identified as an antagonist of Bcl-2, induced by p53.^70, 71, 72^ In view of the fact that more than half of human tumors comprise *p53* mutations, Puma represents an important factor in human cancer as the induction of Puma expression in response to genotoxic anticancer therapeutics is efficiently abrogated in p53-deficient tumors.^73^ Furthermore, ∼40% of primary human BL fail to express detectable levels of Puma and in some tumors this is based on the epigenetic silencing of *puma*.^74^ *puma*^*−/−*^ mice show no abnormalities in hematologic tissue development, whereas lymphocytes, myeloid cells and certain other cell types show resistance to apoptosis induced by growth factor withdrawal or DNA damage.^75, 76, 77^ Suppression of Puma by shRNAs or its genetic ablation in *bim*^*−/−*^ mice enhanced *myc*-driven B-cell lymphomagenesis.^77, 78, 79^ In cooperation with Bim, Puma was additionally shown to be involved in homeostasis of mast cells and macrophages.^77, 80^ Furthermore, the combined deletion of *bim* and *puma*, but not in either single knockout, impaired the elimination of autoreactive T-cells and led to autoimmune reactions in various organs.^81^

After its initial description as a novel phorbol-12-myristate-13-acetate-responsive gene in adult T-cell leukemia, Noxa was rediscovered in a differential display approach using mRNA from γ-irradiated wild-type and *IRF-1/p53* double-deficient mouse embryonic fibroblasts.^82^ Like Puma, Noxa was initially identified as a primary p53-response gene. Noxa has been described as an important determinant of cell death in response to chemotherapy in lymphoid malignancies including CLL, HL, MM and MCL cells.^83, 84, 85, 86^ Furthermore, array-based comparative genomic hybridization and gene-expression microarray analysis showed that Noxa is mutated and preferentially silenced in DLBL.^60^ Gene-targeting experiments of *noxa* in mice displayed defects in T-and B-cell activation and the immune response against viral infection.^87, 88^ Furthermore, Noxa was shown to be centrally involved in neutrophils apoptosis.^89^

By utilizing Bcl-2 protein to screen cDNA libraries the Bcl-xl/Bcl-2-associated death promoter homolog (Bad) was identified.^90^ Bad was the first BH3-only protein to be connected to proximal signal transduction through its differential phosphorylation in response to extracellular survival factors.^91^ In particular, phosphatidylinositol-3-kinase/Akt signaling, a survival pathway frequently hyper-activated in many lymphocytic malignancies, negatively regulates Bad's function.^92^ In line with these observations, *bad*^*−/−*^ mice develop DLBL of the germinal center or post-germinal center, B220^+^CD19^+^ B-cells expressing the zinc finger transcription factor Bcl-6 (latency >15 months).^93^ Although DLBLs were the most frequent tumor entity observed (>40%), *bad*^*−/−*^ mice additionally suffered a broad range of different hematologic malignancies.^93^ This is particularly intriguing, as the inactivation of Bad's pro-apoptotic function by phosphorylation appears to have a prominent role in the survival of these lymphocyte populations.^92^

Together, the data obtained by analyzing human tumor samples and in particular the use of transgenic mouse models conclusively support the notion that the Bcl-2 protein family represents a central regulatory node in the development of hematopoietic system.

## Bcl-2 Targeting as a Therapeutic Option in Hematologic Malignancies

Owing to their imbalance expression levels in tumor cells and their capability to regulate MOMP, Bcl-2 proteins have been viewed as promising therapeutic targets in cancer and research efforts have lately been focusing on the development of drugs targeting Bcl-2 proteins. Accordingly, several small-molecule inhibitors of Bcl-2 proteins have been developed and are currently under clinical evaluation (Figure 3 and Table 1). The following paragraphs summarize the results obtained by surveying these small molecules as a therapeutic option with special emphasis on their activity in hematologic malignancies.

## Bcl-2 Antisense Oligodeoxynucleotide G3139/Oblimersen

G3139 (INN, trade name Genasense; also known as Augmerosen or Oblimersen) is an antisense oligodeoxyribonucleotide, specifically targeting *bcl-2* mRNA. Initial *in vitro* studies using an 18-base phosphorothioate oligonucleotide complementary to the first six codons of the *bcl-2* mRNA (G3139)^94^ showed that this molecule selectively and specifically inhibits Bcl-2 expression in the SU-DHL-4 t(14;18)-containing lymphoma cell lines.^94, 95^ By performing rigorous efficacy, pharmacokinetic and toxicity studies using a number of different lymphoma mouse models^95, 96, 97^ the preclinical evaluation of G3139 was completed and further studies were extended into a phase I study for lymphoma patients with high Bcl-2 expression.^98^ Overall the human phase I studies with G3139 demonstrated good efficacy with low toxicity. In particular, tumor regression, improvement in the laboratory parameters, and symptom improvement together with downregulation of the target protein expression were achieved. Based on these promising results several phase II/III clinical trials were initiated (Table 1). A phase II trial of oblimersen sodium as a single agent showed only modest clinical activity in heavily pre-treated patients with advanced CLL.^99^ However, a separate phase III study of fludarabine plus cyclophosphamide with or without oblimersen showed a 5-year survival benefit in a *post hoc* analysis of patients with CLL who achieved complete (CR) or partial remission (PR).^100^ Oblimersen in combination with rituximab was tested in a phase II trial in non-Hodgkin lymphoma. This study revealed a CR in 23% patients, a PR in 19% patients and 28% of patients showed a minimal response or a stable disease (SD).^101^ Another phase II trial was conducted for the treatment of AML with a combination of oblimersen and gemtuzumab ozogamicin with 10% patients achieved a CR and 15% patients achieved a PR.^102^ Oblimersen was also tested in combination with dexamethasone and thalidomide in a phase II trial for the treatment of relapsed MM patients. Fifty five percent of patients had objective responses, including 2/36 CRs, 4/36 near CRs, and 12/36 PR and 6/36 patients had minimal responses.^103^ However, a randomised phase III trial of oblimersen in combination with other drugs in advanced MM, melanoma or prostate cancer did not show a statistical difference in overall survival.^104, 105, 106^ Based on these results oblimersen was not approved as a therapeutic option by the FDA.^107^ Though, oblimersen efficiently downregulated Bcl-2 in cell culture and thereby effectively reduced lymphoma cell survival the lack of efficiency in primary tumors might be a result of insufficient drug delivery in patients.^108^ Furthermore, those studies demonstrated a long-term benefit for some patients after completing the study and encouraged a renewal of the drug approval, which is still ongoing.

## Gossypol

Gossypol, a natural phenol derived from the cotton plant, was characterized as a specific antagonist of Bcl-xl.^109^ Further studies showed that gossypol acts as a pan-Bcl-2-family inhibitor, capable of binding and inhibiting most antiapoptotic Bcl-2-family members.^110^ Preclinical evaluation of gossypol revealed a potent anti-tumor activity by activating the mitochondrial apoptotic pathway in DLBL,^111^ CML (chronic myeloid leukemia),^112^ CLL,^113^ and non-Hodgkin lymphoma.^114^ Based on these analyses an orally-bioavailable enantiomer of gossypol, AT-101, was evaluated in phase II clinical trials for the treatment of prostate and lung cancer as single agent or in combination with conventional chemotherapeutics (Table 1). However, these efforts did not show convincing clinical activity.^115, 116, 117, 118, 119^ Notably, the most common toxicity was observed in hematologic compartment, suggesting a more potent toxic effect on hematologic malignancies.^115^ Phase II trials for the treatment of hematological malignancies are still in progress.^120^

## Obatoclax (GX15-070)

Obatoclax, a pan-Bcl-2-family inhibitor, was developed from a natural lead compound that potently disrupted the interaction of members of the Bcl-2-family in a functional screen.^121^ As a single agent, obatoclax possesses pronounced anticancer activity in cell lines or primary cells derived from patients suffering from different hematologic malignancies including AML,^122^ mast cell leukemia,^123^ ALL^124^ CLL,^125^ MM,^126^ and HL (Hodgkin Lymphoma).^127^ Albeit promising results were obtained *in vitro* and *in vivo* mouse models, phase I clinical trials for the treatment of AML, CLL, ALL, lymphoma and solid tumors as well as phase II clinical trials for the treatment of HL and myelofibrosis revealed a clinical activity as single agent only in a minority of patients (Table 1).^128, 129, 130, 131, 132^ However, further phase I trials for the combined treatment show very promising clinical activity of obatoclax in specific treatment protocols, such as the combination with bortezomib in MCL^133^ and MM^134^ with 3/12 and 4/10 patients showing a clinical response, respectively.

## ABT-737/ABT-263 (Navitoclax)/ABT-199 (GDC-0199)

A high-throughput NMR-based method was used to screen a chemical library to identify small molecules that bind to the hydrophobic BH3-binding groove of Bcl-xl. The resultant compound ABT-737, developed by Abbott Laboratories (North Chicago, IL, USA), bound with high affinity (Ki ≤1  nM) to Bcl-xl, Bcl-2 and Bcl-w, but not to the less-homologous proteins Bcl-b, Mcl-1 and A1.^135^ Further evaluation of its anti-tumor activity showed that ABT-737 displayed potent single-agent activity against a subset of cell lines representing lymphoid malignancies.^135^ In combination with conventional chemotherapeutics, ABT-737 was shown to potently induce apoptosis in HL,^136^ MM,^137^ AMC,^138^ and CLL ^139^ and also solid tumors such as non-small cell lung cancer (NSCLC).^135^ In addition to established human tumor cell lines, ABT-737 effectively induced apoptosis in primary patient-derived lymphoma and CLL cells *ex vivo*.^135^

However, the prospects for ABT-737 as a therapeutic agent have been hampered by its poor physiochemical and pharmaceutical properties. This compound is not orally bioavailable and its low aqueous solubility makes formulation for i.v. delivery challenging. However, the impressive biological activity of ABT-737 encouraged research to develop an orally-bioavailable compound. On the basis of various pharmacokinetic/pharmacodynamic models and animal studies ABT-263 (Navitoclax; Figure 3), an orraly-bioavailable Bad-like BH3 mimetic (Kis of <1 nM for Bcl-2, Bcl-xl, and Bcl-w) was generated. ABT-263 disrupts Bcl-2/Bcl-xl interactions with pro-death proteins (e.g. Bim), leading to the initiation of apoptosis within 2 h post treatment.^140^ Initial analyses showed that the oral administration of ABT-263 alone induces complete tumor regressions in xenograft models of small cell lung cancer (SCLC) and ALL. In xenograft models of aggressive B-cell lymphoma and MM ABT-263 promoted significant efficacy of clinically relevant chemotherapeutic regimens.^140^ A detailed activity screen revealed that ABT-263 enhances the response of multiple chemotherapeutic regimens, for example, rituximab, rapamycine, rituximab–cyclophosphamide-adriamycin-vincristine-prednisone, and bortezomib, in several models of hematologic malignancies.^141^ However, owing to the crucial role of Bcl-xl in platelet homeostasis^34^ the therapeutic use of ABT-263 was associated with transient thrombocytopenia in preclinical trials.^142^

Based on the results obtained by ABT-737 and ABT-263, Abbott Laboratories has recently developed a high-affinity Bcl-2-selective BH3 mimetic, ABT-199 (GDC-0199), which spared human platelets *in vitro* and dog platelets *in vivo*.^143^ Tumor regression was achieved for xenografts of human lymphoma cell lines. ABT-199 was as effective as ABT-737 in prolonging survival of immuno-competent mice bearing aggressive progenitor cell lymphomas (derived from bitransgenic *myc/bcl-2* mice) without causing thrombocytopenia.^144^ Furthermore, ABT-199 was identified as a promising therapeutic option for the treatment of t(11;14) MM^145^ and AML.^146^ More strikingly, the first clinical trial using a single dose of ABT-199 in three patients with refractory CLL resulted in a rapid tumor lysis within 24 h in 3 of 3 patients.^143^

Together the data obtained by using different pharmacological inhibitors of Bcl-2 indicated a marked susceptibility of hematologic malignancies towards the Bcl-2-targeting protocols (Table 1).

## Perspectives and Restrictions

There are not many anticancer therapeutics that are as exclusively characterized as Bcl-2-targeting strategies concerning their specificity toward the designated target and the mode of their interference with cellular actions and cell death. This is indeed based on our increasing knowledge about Bcl-2 protein family in the last 20 years, which finally has entered the translational stage in the last couple of years. One major aspect about Bcl-2 proteins is their crucial role in the development and the homeostasis of cells of hematopoietic origin. Previous data showed that an imbalanced Bcl-2 protein level causally determines hematologic malignant progression and accordingly targeting the Bcl-2 protein family has been proven to be successful, in particular, in hematologic malignancies. However, manipulations in their function or abundance in the healthy hematologic system may result in fatal changes in this tissue compartment.^147^ Accordingly, this may indicate that in non-hematologic malignancies Bcl-2-targeting strategies should be used with specific caution concerning the functionality and homeostasis of the hematologic system. Alternatively, given the cell type-specific role of some Bcl-2-family members and a more abundant role of other members a highly selective targeting strategy is necessary to avoid severe on-target side effects in the development of other tissues. However, agents targeting the Bcl-2 protein family have been generally shown to be a potent killer of tumor cells derived from hematologic malignancies and accumulating evidence supports the idea that the treatment of other cancer entities may strongly benefit from the Bcl-2-antagonizing protocols in combination with other chemotherapy regimens. The knowledge of how and in combination with which additional chemotherapeutics Bcl-2-antagonizing protocols provoke a potent anticancer effect will strongly foster the clinical evaluation of Bcl-2-targeting strategies.

## Figures and Tables

**Table 1:** Published phase II/III clinical trials of drugs targeting the Bcl-2 family

**Study description**	**Tumor entity**	**Study summary**	**Reference**
Phase II trial of oblimersen as a single treatment	advanced CLL	2/26 patients achieved PR; 7/17 patients showed ≥50% reduction in splenomegaly; 2/7 patients showed complete disappearance of hepatomegaly; 7/22 patients showed ≥ 50% reduction of lymphadenopathy; 11/22 patients showed ≥50% reduction in circulating lymphocyte count	O'Brien *et al.*^99^
Phase III trial of fludarabine plus cyclophosphamide with (group 1) or without (group 2) oblimersen	Relapsed or refractory CLL	17 % CR in group 1 *versus* 7% CR in group 2. Among patients with CR, response duration was significantly longer in group 1 *versus* group 2 (>36 months *versus* 22 months); 40% of patients with CR or PR of group 1 showed a significant 5-year survival benefit	O'Brien *et al.*^128^
Phase II trial of oblimersen in combination with rituximab	Recurrent B-cell non-Hodgkin lymphoma	CR in 23 % patients, a PR in 19 % patients and 28 % patients showed a minimal response or stable disease	Pro *et al.*^101^
Phase II trial of oblimersen in combination with gemtuzumab ozogamicin	AML	12/48 patients (25%) achieved a major response with 5 CR and 7 CR without platelet recovery. Ten of the 12 patients who achieved a major response survived >6 months compared with six of 36 nonresponders	Moore *et al.*^102^
Phase II trial of oblimersen in combination with dexamethasone and thalidomide	Relapsed MM	55% of patients achieved objective responses, including CR in 2/33 patients, 4/33 near CRs, PR in 12/33 patients and 6/33 patients had minimal responses	Badros *et al.*^103^
Phase III trial of dexamethasone with (group 1) or without oblimersen (group 2)	Advanced MM	No significant differences between the two groups in time to tumor progression or objective-response rates	Chanan-Khan *et al.*^105^
Phase II trial oblimersen in combination with dacarbazine	Advanced melanoma	The addition of oblimersen to dacarbazine yielded a trend toward improved survival at 24-month minimum follow-up (median, 9.0 *versus* 7.8 months; *P*=077) and significant increases in progression-free survival (median, 2.6 *versus* 1.6 months; *P*<001), overall response (13.5 *versus* 7.5% *P*=007), complete response (2.8 *versus* 0.8%), and durable response (7.3 *versus* 3.6% *P*=03)	Bedikian *et al.*^104^
Phase II trial of docetaxel in combination with oblimersen	Castration-resistant prostate cancer	No statistical difference in overall survival	Sternberg *et al.*^106^
Phase I/II trial of gossypol in combination with topotecan	Relapsed and refractory SCLC	No convincing clinical activity	Heist *et al.*^115^
Phase II trial of gossypol in combination with docetaxel	NSCLC	No convincing clinical activity	Ready *et al.*^117^
Phase II trial of gossypol	Chemotherapy-sensitive recurrent extensive-stage SCLC	No observed clinical activity	Baggstrom *et al.*^118^
Phase II trial of docetaxel plus prednisone in combination with gossypol	Metastatic castration-resistant prostate cancer	No statistical difference in overall survival	Sonpavde *et al.*^119^
Phase II trial of obatoclax mesylate	Myelofibrosis	No convincing clinical activity	Parikh *et al.*^132^
Phase II trial of Abt-263	Relapsed SCLC	PR in 2.6% and stable disease in 23% patients. The most common toxicity associated with navitoclax was thrombocytopenia, which reached grade III–IV in 41% of patients.	

Abbreviations: AML, acute myeloid leukemia; CLL, chronic lymphocytic leukemia; CR, complete remission; MM, multiple myeloma; NSCLC, non-small cell lung cancer; PR, partial remission; SCLC, small cell lung cancer

## Abbreviations

AML: acute myeloid leukemia
ALL: acute lymphoblastic leukemia
BL: Burkitt's lymphoma
CGH: comparative genomic hybridization
CLL: chronic lymphocytic leukemia
CML: chronic myeloid leukemia
CR: complete remission
DLBL: diffuse large B-cell lymphoma
FDA: Food and Drug Administration
HL: Hodgkin's lymphoma
HSC: hematopoietic stem cells
IMS: mitochondrial intermembrane space
MCL: mantle cell lymphoma
MM: multiple myeloma
MOMP: mitochondrial outer membrane permeabilization
PR: partial remission
SCLC: small cell lung cancer
SD: stable disease
SLE: systemic lupus erythematosus
